# A Multi-Feature Fusion-Based Two-Stage Method for Airport Crater Extraction from Remote Sensing Images

**DOI:** 10.3390/e27121259

**Published:** 2025-12-16

**Authors:** Yalun Zhao, Derong Chen, Jiulu Gong

**Affiliations:** School of Mechatronical Engineering, Beijing Institute of Technology, Beijing 100081, China; 3220205033@bit.edu.cn (Y.Z.); cdr@bit.edu.cn (D.C.)

**Keywords:** crater extraction, multi-feature, image processing, damage information extraction

## Abstract

The accurate extraction of damage information around airport runways is crucial for the rapid development of subsequent damage effect assessment work and the timely formulation of the ensuing operational plan. However, the presence of dark interference areas such as trees and shadows in the background, as well as the increased irregularity at the edge of the crater due to the proximity to the crater, pose challenges to the accurate extraction of the crater area in high entropy images. In this paper, we present a multi-feature fusion-based two-stage method for airport crater extraction from remote sensing images. In stage I, we designed an edge arc segment grouping and matching strategy based on the shape characteristics of craters for preliminary detection. In stage II, we established a crater model based on the regional distribution characteristics of craters and used the marked point processing method for crater detection. In addition, during the step of calculating the magnitude of the edge gradient, we proposed a near-region search strategy, which enhanced the ability of the proposed method to accurately extract craters with irregular shapes. In the test images, the proposed method accurately extracts craters located around and within the runways. Among them, the average recall *R* and precision *P* of the proposed method for extracting all craters around the airport runways reached 89% and 87%, respectively, and the average recall *R* and precision *P* of the proposed method for extracting craters inside the runways reached 94% and 92%, respectively. Meanwhile, the results of comparative tests showed that our method outperformed other representative algorithms in terms of both crater extraction recall and extraction precision.

## 1. Introduction

With the rapid development of military power in various countries, air strikes have become the preferred form of warfare in contemporary high-tech conditions, and airports, as an important support, enabling the air force to carry out air strike missions, are the primary targets for both sides. Therefore, the destruction of enemy airfields is one of the most important combat operations for both sides of a conflict. Over the years, extensive research has been conducted on the destruction of airport runways by various weapons [1,2]. Blockades are an important sign of runway damage, and craters are an important factor affecting blockades [3]. Multiple rounds of missile strikes can effectively block areas such as taxiways, runways, and aprons, which have the functions of enabling passage, takeoff, and landing. Therefore, accurate extraction of craters in remote sensing images is an important prerequisite for assessing the effect of damage on airport targets and enhancing the convenience of target damage effect assessment [4,5,6].

After a round of missile strikes on an airport, it is necessary to extract the craters (generated by explosions) and other damage information within the target range in a timely manner in order to quickly assess the damage effect on targets such as airport runways and formulate further combat plans. The extraction of bomb craters is a challenge due to the small, dark areas presented by the craters in high entropy images, which are easily disturbed by other small, dark areas surrounded by brighter pixels, such as trees and shadows in the background. From the perspective of information theory, these interferences increase the local uncertainty of the image, namely image entropy. In recent years, some scholars have carried out research related to crater detection and achieved various results. According to the different research application backgrounds, the existing crater detection methods are roughly divided into two categories: war legacy crater detection [7,8,9,10,11,12,13,14,15] and crater detection [16,17,18,19,20,21,22,23,24,25,26,27,28]. Among them, studies related to the detection of craters left over from war are mainly used to estimate the distribution of unexploded ordnance in order to reduce the potential risk of war remnants. This paper categorizes studies related to the detection of craters left over from war into two categories: crater detection methods based on supervised learning [7,8,9,10,11] and active detection methods based on marker point processing [12,13], circular features [14,15], etc. Among them, feature-based active detection methods mainly analyze the features of the crater area and then conduct crater detection directly. For example, the authors of [12] present a crater detection method based on marked point processing. The method takes a circular template as the benchmark and uses features such as the high gradient amplitude of the circular edge of the crater and the consistency of the grayscale distribution within the circular crater area to establish a crater model in the form of an energy function. Then, the detection of all craters within the target range is achieved by combining the Reversible Jump Markov Monte Carlo method with the simulated annealing method for global optimization. The authors of [14] simplify the craters to circular regions and propose a circular detection algorithm for detecting craters. The authors of [15] present a method for extracting craters within airport runways based on multi-feature fusion. The method first extracts the complete runway areas. Then, based on the runway extraction results, the edge gradient amplitudes of the candidate circular regions and the grayscale distribution consistency within the regions are calculated within the runway, and crater detection is carried out through a fixed threshold. This method achieves a relatively accurate extraction of crater areas located within the runway. The crater extraction effect of the second type of method depends directly on the accuracy of the crater model. The authors of [12,14] both make significant simplifications to the crater model, and there is relatively little interference in their test images. The authors of [15] only consider the extraction of crater areas located inside a runway, and thus, their crater model is designed relatively simply. At the same time, proximity to craters or additional damage may increase the irregularity of the crater edge shape, resulting in a reduction in the geometric characteristics and roundness of the continuous edge and increasing the difficulty of crater detection. In addition, the application of existing crater detection methods is limited when there is a lot of interference from other small, dark areas, such as trees and shadows, in the background of the image. For supervised learning-based craters, their application in airport crater extraction is limited due to the lack of a large dataset of manually labeled post-damage airport crater distribution images.

Another category of research related to crater detection is mainly applied in scenarios such as stellar lifetime studies, spacecraft landing, and navigation. Craters and war legacy craters have similar shape characteristics. Craters are usually formed by the impact of meteoroids, and early war legacy crater detection methods were borrowed from crater detection research. The research related to crater detection in this paper is roughly divided into feature-based active detection methods [16,17,18,19,20,21,22] and sample data-driven crater detection methods [23,24,25,26,27,28]. The first category, that of feature-based active detection methods, mainly involves analyzing the characteristics of the crater areas, establishing a crater model, and then conducting crater detection directly. Among them, the authors of [16] presented a crater detection method based on the Hough transform at the midpoint of the chord. This method first combines edge detection and point detection to generate rectangular candidate regions containing craters. Then, using the circular features of craters, the midpoint of the crater is detected within the candidate region through the Hough transform of the midpoint of the chord to determine the midpoint position of the crater, thereby achieving the detection of the crater. The authors of [17,18] use the morphological characteristics of craters to detect craters by constructing circular binarization templates in a template-matching manner. The authors of [19,20] use the shadow and light regions formed by concave craters under the sun to align these features with the corresponding shadow and light regions that are typical of a crater through segmentation. Then, the craters are precisely located and detected by fitting the edges to obtain circular areas. To sum up, in the current applications of these crater detection methods, the image background is relatively pure, and there are fewer areas of interference. At the same time, the existing crater detection methods rely more on the circular features of the craters. The second category of craters, driven by sample data, includes machine learning methods such as deep learning [23,24,25,26], decision trees [27], and support vector machines [28]. The application of these methods relies on large sample datasets and time-consuming, manually labeled data.

In recent years, due to the powerful performance of convolutional neural networks (CNNs) in remote sensing and image processing, deep learning methods have been widely developed in the field of remote sensing and image processing. For the popular field of object detection, there are currently classic two-stage detectors such as RCNN [29] and Faster R-CNN [30], which first generate candidate regions and then classify and refine each region. These methods have high detection accuracy but slow speed. Other classic single-stage detectors, such as YOLO [31,32], treat detection as a classification problem and are extremely fast. In addition, transformer architecture [33] shows significant advantages in tasks that require global contextual information and complex scene processing. Considering the dark, small areas presented by craters in images, some scholars have conducted relevant research on small object detection based on deep learning methods [34,35,36,37,38] and achieved good detection results. For example, to improve the accuracy of detection results achieved for small targets, the authors of [35] propose a new small target detection model, Enhanced-YOLOv8. This method utilizes a small target detection level (STDL) to achieve more accurate target localization and bounding box accuracy. These authors propose a fusion convolutional block attention module (FCBAM) to amplify global dimensional features and enhance the feature fusion capability, with a semantic fusion network (SFN) used to compensate for the absence of spatially detailed information in the high-layer feature. The authors of [37] utilize deep feature learning and a feature fusion network (DFLFFN) to help detect objects. In addition, regarding the noise present in large-scale datasets, the authors of [39] propose a progressive sample selection framework with contrastive loss for noisy labels, named PSSCL. Regarding the poor quality of underwater images, the authors of [40] propose a content-style control network with style contrastive learning (CSC-SCL). This type of deep learning method achieves good detection results in the field of detection, but it relies on large sample datasets and time-consuming, manually labeled data. Therefore, due to the scarcity of data, deep learning methods are difficult to apply to the application scenario in this paper.

Therefore, summarizing the limitations of the existing crater detection methods while considering the significant features of craters in high entropy images, this paper combines multiple features such as the edge shape features of the crater area, the high gradient amplitude features of the edge, the size of the crater area, and the gray distribution of the area inside the crater to propose a multi-feature fusion-based two-stage method for airport crater extraction from remote sensing images. Inspired by the theory of information entropy, this method fully utilizes the local features presented in high entropy images to achieve accurate extraction of damage information. First, based on the overall high luminance characteristics of an airport runway and the surrounding areas, we extract the target area, containing the airport runway, to reduce the interference of the background area. Then, in the first stage, a set of candidate regions is generated within the target region using a blob detector, and a candidate region screening method based on continuous-edge arc-segment group matching is designed to screen the candidate regions in order to reduce the search space for crater extraction. Finally, in the second stage, the crater detection model was constructed by combining the edge high-gradient-amplitude feature of the craters with the regional distribution feature inside the crater, and the Reversible Jump Markov Chain Monte Carlo sampling method (RJMCMC) was combined with the simulated annealing algorithm to achieve accurate extraction of crater regions. Finally, we verified the effectiveness of the method proposed in this paper through experiments.

The remaining sections of this article are arranged as follows. In Section 2, we elaborate on the method of extracting craters from remote sensing images that is proposed in this paper. In Section 3, we first present the dataset and evaluation metrics; then, we show the experimental results of the method proposed in this paper and compare them with the existing typical representative methods and analyze the experimental results. In Section 4, we discuss the key strengths and difficulties of the method proposed in this paper. In Section 5, we summarize the method proposed in this paper and future works.

## 2. Methodology

**Figure 1:**
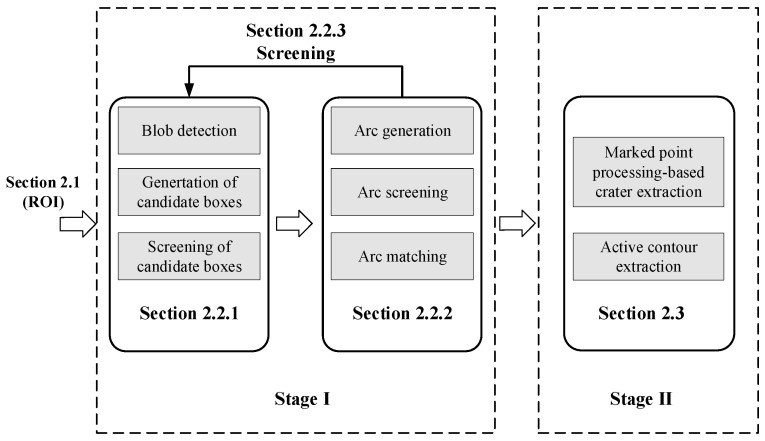
Flowchart of the proposed crater extraction method.

The content of the crater extraction method proposed in this paper mainly includes the extraction of the target area of interest, the generation and screening of candidate areas, and the detection and extraction of crater areas. In this section, we will provide a detailed introduction to the crater extraction method proposed in this paper. The overall process framework of the crater extraction method proposed in this paper is shown in Figure 1. In stage I, the craters’ characteristics, such as a near-circular shape, size, and low brightness, are utilized. In stage II, the characteristics of high gradient magnitude at the edge, consistency of grayscale distribution, and divergent distribution from the center outward are utilized.

### 2.1. Extraction of Region of Interest

To reduce the interference of the background region and improve the accuracy of crater detection, here we use the adaptive-threshold fuzzy-enhancement threshold segmentation algorithm designed in our previous work [41] to preprocess the input image. By taking advantage of the high luminance characteristics of the airport runway area as a whole, the target area of interest, including the airport runway, is extracted (the smallest envelope rectangle of the largest connected area after segmentation). The area of the envelope rectangle is extracted to generate the region of interest, and this region is used as the input of the crater extraction method proposed in this paper.

First, a Gaussian filter is performed on the input image to reduce noise interference. Construct a membership function *F*, and calculate the membership of every pixel’s gray value in the filtered image to obtain the fuzzy set *L*:

(1) $$L_{i,j}=F(I_{i,j}),L_{i,j}\in [0,1]$$

where $I_{i,j}$ represents the gray value of a pixel (i,j) within the range of [0–255], $L_{i,j}$ is the mapping of the membership function.

Then, smooth the statistical distribution curve of the grayscale histogram with a Gaussian filter, and calculate the dividing threshold *Thr* of the fuzzy set, adaptively, according to the extreme value distribution characteristics. Based on the dividing line, the calculation of enhancement processing can be expressed as

(2) $${\hat{L}}_{i,j}=\left\{\begin{array}{l} Thr\times L_{i,j}^{2}\;\;\;\;\;,\;0\leq L_{i,j}<Thr\\ 1-Thr\times {\left(1-L_{i,j}\right)}^{2}\;,\;Thr\leq L_{i,j}\leq 1 \end{array}\right.$$

where ${\hat{L}}_{i,j}$ is the corresponding membership degree after fuzzy enhancement processing.

Finally, the result after the enhancement processing is mapped back to the gray value interval [0–255] through the inverse transformation of the membership function. The inverse transformation is expressed as follows:

(3) $${\hat{I}}_{i,j}=F^{-1}({\hat{L}}_{i,j})$$

After the above fuzzy enhancement process, the contrast between the target and the background area is enhanced, and the statistical distribution curve of the gray histogram is closer to the bimodal characteristic, which is precisely the best application scenario of the Otsu method. Then the Otsu threshold segmentation method can effectively distinguish the target and the background. The segmentation threshold is calculated according to the Otsu method. Based on the segmentation threshold, the segmented background pixels are set to zero to obtain the region of interest containing the airport runway:

(4) $${\hat{I}}_{i,j}=\left\{\begin{array}{l} 0\;\;,if\;\;{\hat{I}}_{i,j}\;<\;Th\\ {\hat{I}}_{i,j}\;\;\;,if\;\;{\hat{I}}_{i,j}\;>\;Th \end{array}\right.$$

where *Th* is the segmentation threshold calculated by the Otsu method.

### 2.2. Candidate Crater Region Generation and Screening (Stage I)

#### 2.2.1. Generation of Candidate Crater Regions

We use the blob detector used by the author of [12] to generate a set of initial candidate regions of the crater, given its center coordinates and size, which can be expressed as $\left\{R\right\}$. This initial candidate set limits the search space in the image for subsequent crater detection based on marker point processing. Considering that the edge roundness of the actual crater area may be low and irregular, in order to enable the crater model proposed in this paper to extract any possible crater areas, it is essential that we appropriately select the parameters of the blob detector. Theoretically, as long as the parameters are chosen appropriately, the blob detector can detect craters of any size and shape.

#### 2.2.2. Feature Detection in the Candidate Regions

In this subsection, we take the initial set of candidate regions generated in Section 2.2.1 as input, perform continuous edge extraction within each candidate region, and perform arc segment separation, arc segment screening, and arc segment grouping on the continuous edges. Based on the results of arc screening and grouping, we proceed with the subsequent screening of candidate regions.

(1) Separation of arc segments. First, we perform edge enhancement within the candidate regions, extracting and obtaining a continuous set of edges. Then, using the linear and arc segment separation methods, based on the curvature threshold of the continuous edge, as was used in our previous work [15], we establish the geometric model of the continuous edge, calculate the curvature threshold, and separate the continuous edge based on this threshold (the threshold can be calculated according to the formula $k_{i}=G(\theta_{i},\sigma ,L)=K\cdot \cot(\theta_{i}/2)$ [15]) to obtain the arc segment set of the continuous edge:

(5) $$C=\{c_{m}:(u_{i},v_{i}),m=1,2,\ldots n\}$$

where *m* represents the index of the number of continuous edges within a candidate region, and *i* represents the index of pixels on a continuous edge.

**Figure 2:**
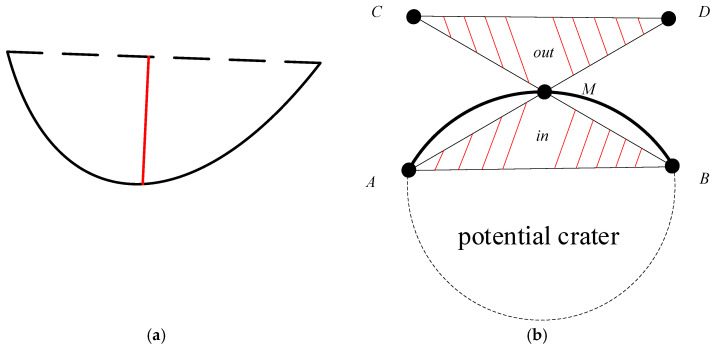
Diagram of the edge arc segment. (**a**) convexity of the arc segment; (**b**) the triangular area inside and outside the arc segment.

(2) Screening of arc segments. After the arc segment separation is completed, the continuous edge arc segments obtained from the separation are screened, and only the edge arc segments with similar geometric properties to the crater edge arc segments are retained to reduce interference and improve the accuracy of crater detection. According to the shape and low luminance characteristics of the crater areas, we designed the screening strategy with the following aspects: (a) Length constraint. Eliminate continuous edge arcs shorter than the given length threshold to reduce the number of short edge arcs, and minimize the interference of messy edges in the background while reducing the computational load. (b) Convexity constraint. As shown in Figure 2a, convexity is defined as the maximum distance from a point on a continuous edge arc to the line connecting the two endpoints of the continuous edge arc, and we eliminate continuous edge arcs with convexity less than the given threshold. (c) Internal and external gray-level difference constraint. As shown in Figure 2b, *A* and *B* are the two endpoints of the arc segment, *M* is the midpoint of the arc segment, and *C* and *D* are the symmetrical points about the endpoints *B* and *A*, respectively. Construct the triangular area Δin as the area located inside the continuous edge arc segment $\overset{⏜}{AMB}$, and the triangular area Δout as the area corresponding to the area Δin located outside the continuous edge arc segment. Usually, the area inside the crater edge arc has a smaller gray value than the area outside the crater, that is, $G_{\Delta in}<G_{\Delta out}$ (where $G_{\Delta in}$ represents the gray value of area AMB, $G_{\Delta out}$ represents the gray value of area CMD), and we eliminate the continuous edge arc that does not satisfy this constraint.

(3) Grouping of arc segments. In candidate regions containing crater areas, we consider that there are mainly three forms of continuous edge arcs in crater regions: closed arcs, single arcs, and arc pairs. A large number of pseudo-crater regions are present in the initial candidate region set generated by the blob detector. We grouped the set of arc segments obtained above within the candidate region based on the different forms presented by the arc segments at the edge of the crater. We will obtain candidate region blocks with closed arc segments, single arc segments, and arc segment pairs, respectively.

**Figure 3:**
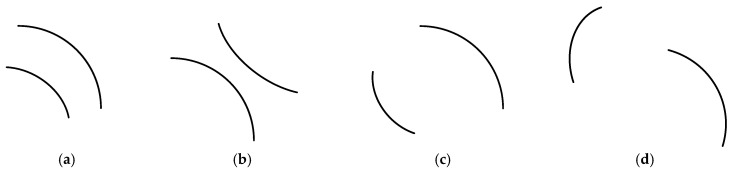
Geometric convexity relationship between arc segment pairs. (**a**) same direction; (**b**) back to back; (**c**) reverse; (**d**) intersection.

Among them, the edge arc segment pairs belonging to the same crater region need to satisfy the geometric convexity constraint. If the convexes of the two arcs *c_i_* and *c_j_* are facing opposite directions, that is, the geometric relationship between the two arcs is “back-to-back”, it can be determined that they do not belong to the same crater edge. Four cases of the geometric relationship between paired arcs are shown in Figure 3 below. Among them, the arc segment pair in Figure 3c satisfies the geometric convexity constraint of the arc segment at the edge of the same crater. The arc segment pair in Figure 3d may correspond to the distribution scene of edge arcs in the contiguous area of multiple craters (adjacent craters), satisfying the geometric convexity constraint of the edge of the irregularly shaped crater area.

**Figure 4:**
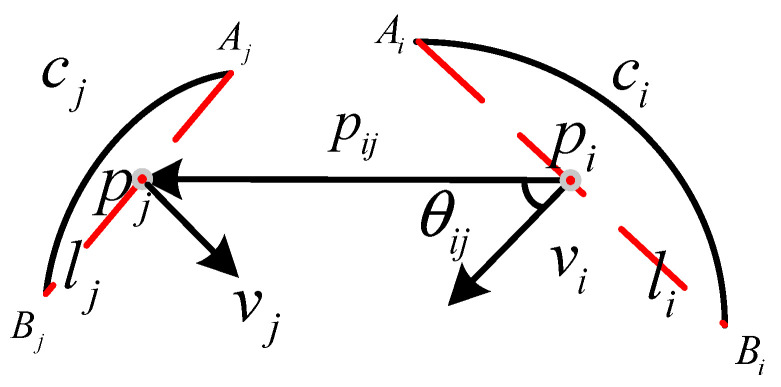
Diagram of the geometric constraints of an arc segment pair.

We adopt the convexity calculation formula used by the author of [42] to calculate the convexity constraints of the arc segment pairs. As shown in Figure 4, *l_i_* and *l_j_*_,_ respectively; represent the line segments formed by the endpoints of the arc segments *c_i_* and *c_j_* as *v_i_* and *v_j_*_,_ respectively; represent the unit normal vectors perpendicular to the chord of the consecutive edge arc segments (in the same direction as the concave surface of the arc segment) as *A* and *B*, respectively; represent the endpoints of *l_i_* and *l_j_* as *p_i_* and *p_j_*_,_ respectively; represent the midpoints of *l_i_* and *l_j_* as θ*_ij_* and θ*_ji_*_,_ respectively; and represent the angles between vectors *v_i_* and $\vec{p_{i}p_{j}}$ as *v_j_* and $\vec{p_{j}p_{i}}$. Then, the condition for the arc segments *c_i_* and *c_j_* needed to satisfy the geometric convexity constraint is

(6) $$\vec{p_{i}p_{j}}\cdot v_{i}>0\;\;\&\;\;\vec{p_{j}p_{i}}\cdot v_{j}>0$$

where

(7) $$v=\left(\frac{\left(A_{j}-B_{j}\right)}{\sqrt{{\left(A_{j}-B_{j}\right)}^{2}+{\left(A_{i}-B_{i}\right)}^{2}}},\frac{\left(B_{i}-A_{i}\right)}{\sqrt{{\left(A_{j}-B_{j}\right)}^{2}+{\left(A_{i}-B_{i}\right)}^{2}}}\right)$$

#### 2.2.3. Screening of Candidate Crater Regions

In this section, we take the initial candidate region set generated in Section 2.2.1 and the edge arc segment set obtained in Section 2.2.2 as input. Based on characteristics such as the edge shape, area size, and the low brightness of the crater areas, the candidate crater regions are filtered using the group matching strategy on the continuous arc segments of the edge to reduce the number of interference regions and further reduce the search range for subsequent crater detection.

First, candidate regions that do not contain the characteristics of the three forms of continuous edge arc segments, as described in Section 2.2.2, are excluded. Then, based on the size of the crater area, the low luminance features of the crater area, and the central minimum features of the area, the screening strategy for the candidate regions is designed based on the set of closed arc, single arc, and arc pairs. Retaining the candidate crater regions that satisfy the feature constraints, a set of candidate regions that may have craters is obtained as $R_{B}\subseteq R$, which can be defined as follows:

(8) $$R_{B}=\left\{R_{i}\in R\left|\;K_{1}(R_{i})\wedge K_{2}(R_{i})\wedge K_{3}(R_{i})\right.\right\}$$

where *K* represents the set of conditions that must be met for candidate region screening. The conditions in the set *K* are defined as follows:

(1) Constraint of the crater area’s low luminance. Using the low luminance characteristics of the crater area, we calculate the gray-level mean sum of the internal $G_{\Delta in}$ and external $G_{\Delta out}$ areas of the continuous edge arc segment, respectively (as shown in Figure 2b). Then, the candidate regions where the continuous edge does not satisfy the internal and external gray-level difference constraints are eliminated. The set of candidate regions that satisfy the low luminance constraint of crater area is obtained:

(9) $$R_{1}=\left\{R_{i}\in R\;\left|\;G_{\Delta in}<G_{\Delta out}\right.,G\;=\;\frac{\sum_{j=1}^{n}p_{j}}{n},\forall p_{j}\in s\right\}$$

where $G_{\Delta in}$ and $G_{\Delta out}$, respectively, represent the mean grayscale value of the inner and outer areas of the candidate crater edge, $p_{j}$ represents the grayscale value of any pixel within the corresponding area, *n* represents the total number of pixels within the corresponding area, and *s* represents the corresponding triangular area. The internal and external areas are shown in Figure 2b.

**Figure 5:**
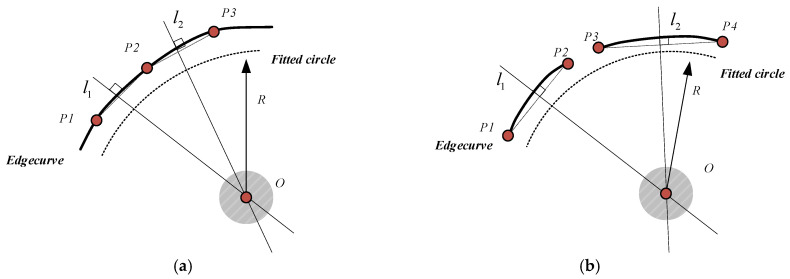
The calculation of center coordinates based on arc segments. (**a**) The single arc segment mode; (**b**) the arc segment pair mode.

(2) Constraint of the crater area’s size. Taking advantage of the fact that the crater area is nearly circular and has a small area, the continuous edge arc segment set extracted in Section 2.2.2 is used to fit the nearly circular area, and the area size is used for screening. According to the arc segment grouping results, the arc segment fitting can be divided into two scenarios: single arc segment fitting and arc segment pair fitting. In the single arc segment scenario, as shown in Figure 5a, three edge points, *P*_1_, *P*_2_, and *P*_3,_ of an arc segment are selected at certain intervals as the endpoints of the chord, and the lines *l*_1_ and *l*_2_ are perpendicular lines passing through the midpoint of chords *P*_1_*P*_2_ and *P*_2_*P*_3,_ respectively. We take the intersection point of lines *l*_1_ and *l*_2_ as the center point *O* of the approximate circle obtained by fitting. The mean of the distance from the center point to the edge arc segment points is taken as the radius *r* of the fitted circular region. As shown in Figure 5b, for the observed arc segment pair, the endpoints of each arc segment are selected as the endpoints of the chord to calculate the center point *O*, and the average distance from the center point to the edge arc segment points is taken as the radius *r* of the fitted circular region to obtain the approximate circular region $R_{f}$. Finally, the corresponding radius threshold is set according to the crater area range to obtain the set of candidate regions that satisfy the crater area’s size constraint:

(10) $$R_{2}=\left\{R_{i}\in R\;\left|\;r\leq T_{r}\right.,r=\frac{\sum_{j=1}^{n}dist(O,P_{j})}{n},P_{j}\in C\right\}$$

where *O* represents the center point of the fitted circle, *P_j_* represents a pixel point in the arc segment point set, *T_r_* represents the radius threshold of the crater area, *n* represents the length of the arc segment point set, and *C* represents the discrete point set of the edge arc segment.

(3) Constraint of the minimum brightness position. Usually, the pixel values at the center of the crater areas in the image have the characteristic of being local minima. Therefore, using the pixel with local minimum brightness, the circular area obtained by fitting in step (2) is used for range constraints. The position of the local minimum pixel *M* in the area is judged to ensure that the minimum point is within the fitted circular area, and a set of candidate regions that satisfy the center minimum constraint is obtained:

(11) $$R_{3}=\left\{R_{i}\in R_{2}\;\left|\;d\leq r,d=dist(M,P_{j}),\forall P_{j}\in Edge_{R_{f}}\right.\right\}$$

where *P_j_* represents any pixel point on the circular edge, *M* represents the local minimum pixel point in the area, *R_f_* represents the fitted circular region, and *dist*(*A*,*B*) represents the Euclidean distance between two points. The straight-line distance of two points can be calculated as follows:

(12) $$dist(A,B)=\sqrt{{\left(A_{x}-B_{x}\right)}^{2}+{\left(A_{y}-B_{y}\right)}^{2}}$$

Through the above steps of constraints and screening, some interference regions that do not contain craters can be effectively eliminated, thereby reducing the number of candidate regions. The set of candidate regions after screening can be expressed as $R_{B}=R_{1}\cap R_{2}\cap R_{3}$. Then, based on the fitting results of the edge arc segments within the candidate regions, the candidate regions are repositioned with the center coordinate *O* of the fitted circular regions as the center. The suspected crater is placed at the center of the candidate region, and uses the eigenvector, consisting of the coordinates of the center point of the region and the radius of the fitting circle, as the marker of the candidate region. The center point coordinates and radius serve as the initial position and reference size for subsequent crater detection, respectively.

### 2.3. Crater Extraction (Stage II)

In this section, we take the candidate region set *R_B_* obtained from the above steps as input and construct a crater detection algorithm framework based on the marked point process (MPP). The marked point process is a combination of the spatial point coordinate $L_{i}=(x_{i},y_{i})$ and the geometric feature marker $m_{i}$ added to the corresponding point to create MPPs through the association between the spatial point coordinates and the corresponding markers. Among them, the coordinate distribution space of the labeled points is limited to $R\subset {\mathbb{R}}^{\nu }$, where ℝ represents the input two-dimensional digital image data; therefore, ν = 2. The main idea of the marked point process is to model objects within a random framework, with the aim of finding a set of objects $\mu^{*}=\left\{\mu_{1},\mu_{2},\ldots ,\mu_{n^{*}}\right\},n^{*}\leq n$ of the same class that satisfy the model constraints in the created set of marked points $\mu =\left\{\mu_{1},\mu_{2},\ldots ,\mu_{n}\right\}$, through which the probability density function of the model is maximized.

**Figure 6:**
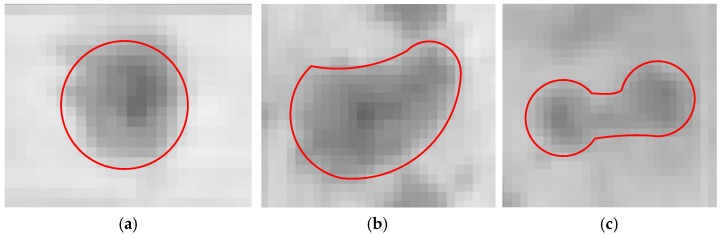
Example of craters with irregular shapes in image 1. (**a**) Circular crater; (**b**) irregular crater; (**c**) adjacent craters.

In this paper, the probability density function finds craters with irregular shapes (as shown in Figure 6b,c) in the form of Gibson energy $\exp^{-U\left(\cdot \right)}$ in combination with the characteristics presented by the crater region in the image [12]. Its optimal configuration is achieved by minimizing the Gibson energy (maximizing the probability density function), that is, $\mu^{*}=\arg\min U\left(\cdot \right)$. Meanwhile, we combine the Reversible Jump Markov Monte Carlo method (RJMCMC) [43] with the simulated annealing method to search for marked points with the same type of object configuration in order to obtain the optimal configuration that satisfies the constraints of the crater model. In the sampling process, we use the set of candidate regions *R_B_* to limit the search space in the image and provide information on the center coordinates and initial radius of the candidate craters. Among them, the high gradient amplitude of the actual edge of the crater and the grayscale distribution characteristic inside the crater are beneficial for reducing the energy of the crater model, and the search results are a set of crater objects detected in each image.

#### 2.3.1. Establishment of Crater Model

The author of [12] obtained a final detection result of a single target by independently detecting multiple images of the same targets and combining the detection results of multiple images of the same target to utilize the redundant information. This method requires multiple overlapping images of the same target and the conduction of search detection on each image, which results in a doubling of the detection time. Meanwhile, the proposed crater detection algorithm models craters in a circular form and constructs an energy function model based on the high gradient amplitude of the circular edge of the crater, the consistency of grayscale within the crater area, and the non-overlapping characteristics between craters. In this model, the gradient amplitude at the crater edge is calculated as the component of the circular edge pixel points pointing towards the center of the circle, and the adjacent craters are modeled as penalty terms. With this model, it is difficult to effectively detect craters with poor roundness or irregular shapes (as shown in Figure 6b,c).

In this paper, we summarize the characteristics of the crater area presented in the image. The craters are modeled by combining the high gradient amplitude characteristics at the edge of the craters, the consistent grayscale distribution characteristics within the craters, the regional minimum grayscale at the center of the craters, and the divergent grayscale distribution from the center to the edge. The crater model can be represented as follows:

(13) $$U=U_{g}+U_{h}+U_{a}$$

where $U_{g}$ checks the high edge gradient magnitude characteristic of the crater area, $U_{h}$ checks the grayscale consistency within the crater area, and $U_{a}$ checks the grayscale divergence distribution characteristics of the crater area.

First, a circular reference template for the crater area is constructed with the center point coordinate of the candidate region as the center and the radius *r* as the radius. The circular template can be represented as follows:

(14) $$M_{c}={\left[\begin{array}{ccccc} \left(-1,0\right) & \left(0,1\right) & \ldots & \left(1,0\right) & \left(0,-1\right)\\ \left(-2,0\right) & \left(0,2\right) & \ldots & \left(2,0\right) & \left(0,-2\right)\\ \begin{array}{l} \vdots \end{array} & \begin{array}{l} \vdots \end{array} & \begin{array}{l} \ddots \end{array} & \begin{array}{l} \vdots \end{array} & \begin{array}{l} \vdots \end{array}\\ \left(-r,0\right) & \left(0,r\right) & \ldots & \left(r,0\right) & \left(0,-r\right) \end{array}\right]}_{r\times 8}$$

where the eight component elements $M_{c}(i,j),i=1,2,\ldots ,r;j=1,2,\ldots ,8$ in each row of the template matrix represent eight pixels on a crater ring of equal radius and equal interval, the component elements in each column of the template matrix represent *r* evenly spaced pixels along the radial direction. Based on this reference template matrix, it is convenient to calculate characteristic quantities, such as the gradient magnitude at the crater edge, the grayscale distribution consistency inside the crater area, and the grayscale divergence distribution of the crater area.

**Figure 7:**
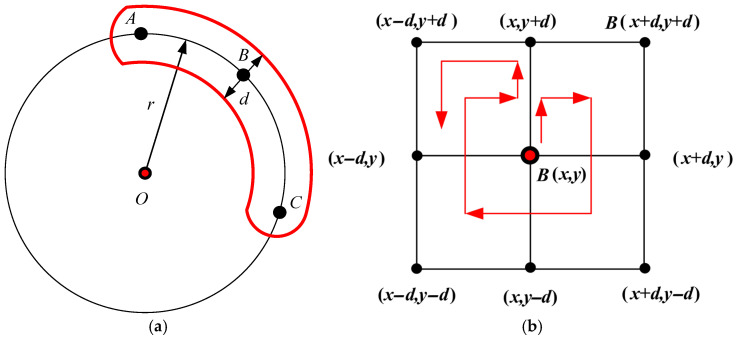
Illustration of crater edge search (*d* indicates the search neighborhood radius). (**a**) Crater circular reference template (*O* indicates the center of the crater); (**b**) neighborhood of circular edge pixel.

(1) High gradient magnitude at the edge. Due to the weak light reflection intensity in the inner area of the crater, the gradient amplitude in the transition region at the crater edge often has the characteristic of being a local maximum. Here, we adopt a near-region search strategy to calculate the gradient values of actual edge points of irregularly shaped craters. Based on the center coordinates and initial radius parameters provided by the candidate regions obtained in Section 2.2.3, the region search is conducted within the neighborhood of the radius d=r∗He with the edge pixel point $M_{c}(r,i)$ of the crater’s circular reference template as the center (as shown in Figure 7a,b). Calculating the gradient amplitudes of the real edge points of the crater with the maximum gradient magnitude within the region and counting the gradient amplitudes of all edge pixels of the candidate craters can improve the ability of the established crater model to detect craters with irregular shapes and poor roundness. Based on the calculation of the actual edge gradient amplitude of the crater, the actual edge gradient amplitude model of the crater (the first term *U_g_* of the energy model in Equation (9)) can be established as follows:

(15) $$\begin{array}{l} U_{g}=f_{g}\sum_{\mu_{i}\in X_{t}}\left(T_{g}-\frac{1}{n}\sum_{j=1}^{n}\nabla_{Edge_{pj}}^{⊥}\right),\\ \nabla_{Edge_{pj}}^{⊥}=\max(\nabla_{p_{i}}^{⊥},\forall p_{i}\in \overset{\circ }{U}(p_{j},d),p_{j}=M_{c}(r,j)) \end{array}$$

where $\nabla_{Edge_{pj}}^{⊥}$ represents the magnitude of the gradient from the actual edge pixel of a single crater to the center of the crater, *n* represents the total number of pixels involved in the calculation at the actual edge of a single crater, $\nabla_{p_{i}}^{⊥}$ represents the gradient amplitude of all pixels in a neighborhood of radius *d* with the edge pixel $p_{j}$ of the circular reference template as the center towards the center of the crater, $\overset{\circ }{U}(p_{j},d)$ represents the neighborhood with $p_{j}$ as the center and *d* as the radius, $f_{g}$ represents the weighted factor coefficient of the term in the energy model, $X_{t}$ represents the set of craters in the *t*-th state, and $\mu_{i}$ represents the *i*-th crater in the set of craters. The parameter $T_{g}$ is set to ensure that the high gradient amplitude at the edge of the crater in the same state can reduce the overall energy.

(2) Consistency of grayscale distribution. Affected by factors such as weak light reflection intensity, the overall brightness within the crater area is low, and the grayscale distribution is relatively uniform within the circular area of the same radius. Uniformity is measured by calculating the standard deviation of grayscale values within the area. Usually, the calculated standard deviation of the grayscale value in the disturbed area is higher than the standard deviation of the grayscale value in the inner area of the real crater. By defining the standard deviation threshold *T_h_* as a constraint, the standard deviation term of the real crater area can reduce the overall energy value of the model. The establishment of the grayscale distribution consistency model for the crater area (the second term *U_h_* of the energy model in Equation (9)) can be performed as follows:

(16) $$\begin{array}{l} U_{h}=f_{h}\sum_{\mu_{i}\in X_{t}}\max\left(0,\sigma_{i}-T_{h}\right)\;,\\ \sigma_{i}=\sum_{j=1}^{r}\sigma_{j}\;,\;\sigma_{j}=\sqrt{\frac{1}{N}\sum_{o=1}^{N}\left(p_{o}-\mu \right)}\;,\;p_{o}\in M_{c}(j,:) \end{array}$$

where $\sigma_{i}$ represents the standard deviation of the grayscale distribution of all pixels within the crater area, $\sigma_{j}$ represents the standard deviation of pixels on an equal-radius ring calculated based on a circular reference template $M_{c}$, *N* represents the number of pixels involved in the calculation on the circular ring, $T_{h}$ represents the given threshold of standard deviation, $f_{h}$ represents the weighted factor coefficient of the term in the energy model, $X_{t}$ represents the set of craters in the *t*-th state, and $\mu_{i}$ represents the *i*-th crater in the set of craters.

(3) Divergent distribution of the center outward. The center of the crater, due to the weakest reflected light intensity, usually shows a regional minimum grayscale value in the image, and the grayscale value of pixels gradually increases along the radial direction from the center to the edge of the crater. This distribution is described by the cumulative difference in pixel grayscale values between rings with different radii. The model of the outward divergent distribution of the crater center (the third term *Ua* of the energy model in Equation (9)) can be established as follows:

(17) $$\begin{array}{l} U_{a}=f_{a}\sum_{\mu_{i}\in X_{t}}\max\left(0,\left(T_{a}-\sum_{i=2}^{r}\left(s_{i}-s_{i-1}\right)\right)\right)\;,\;\\ s_{i}=\frac{1}{N}\sum_{j=1}^{N}M_{c}(i,j)\;,\;i=1,2,\cdots ,r \end{array}$$

where $s_{i}$ represents the mean grayscale value of all pixels within the circular ring, *N* represents the number of pixels involved in the calculation on the circular ring, and $f_{a}$ represents the weighted factor coefficient of the term in the energy model. The parameter $T_{a}$ is set to ensure that the energy model component value calculated for real craters can reduce the energy value of the overall crater model, $X_{t}$ represents the set of craters in the *t*-th state, and $\mu_{i}$ represents the *i*-th crater in the set of craters.

#### 2.3.2. State Transformer

Considering the Reversible Jump Markov Monte Carlo method (RJMCMC) [43] can effectively model a sampling problem with an unknown number of target objects, in this paper, RJMCMC is combined with the simulated annealing algorithm to find the best configuration that satisfies the given constraints through iterative wandering among the states. In each iteration, we set three updated states—birth, death, and update—to update the current configuration. Among these, the birth status means an object is randomly selected to participate in the calculation and update the overall configuration. The dead state randomly selects a target object, removes it from the current configuration, and updates the configuration of the current object. The update status randomly selects a crater target object to perform a translation or size change operation on the selected object, recalculates the model value of the updated object, and updates the current configuration. Then, the acceptance rate *α* is calculated and used to judge whether to accept the state of the configuration after the change or to maintain the state of the configuration before the change. The state transition is based on the detailed equilibrium equation of the Markov chain, which can be expressed as follows:

(18) $$\int_{A}\int_{B}\pi^{*}(x)P(x,x^{\prime })\;=\;\int_{B}\int_{A}\pi^{*}(x^{\prime })P(x^{\prime },x)$$

where $\pi^{*}(\cdot )$ represents the stationary distribution of the Markov chain, and *P* represents the state transition matrix when the stationary distribution is reached.

The acceptance rate *α* can be calculated as follows:

(19) $$\alpha (x,x^{\prime })\;=\;\min\left\{\;1\;,\;\frac{\pi^{*}(x^{\prime })Q(x^{\prime },x)}{\pi^{*}(x)Q(x,x^{\prime })}\right\}$$

In this paper, we represent stationary distribution states using the Gibson energy in the form of $\exp^{-U\left(\cdot \right)}$, that is, in Equation (15), $\pi^{*}(\cdot )\propto \exp^{-U\left(\cdot \right)}$. Thus, we transform the problem of finding the maximum probability of a stationary distribution into the problem of finding the minimum Gibson energy. Referring to the authors of [12], we define the acceptance function as follows:

(20) $$\alpha (x,x^{\prime })\;=\;\min\left\{\;1\;,\;\frac{P_{Q_{\left(x,x^{\prime }\right)}^{inv}}}{P_{Q_{\left(x,x^{\prime }\right)}}}\cdot \frac{Q_{\left(x^{\prime },x\right)}\left(x\to x^{\prime }\right)}{Q_{\left(x,x^{\prime }\right)}\left(x^{\prime }\to x\right)}\cdot \exp\left(-\frac{U\left(x^{\prime }\right)-U\left(x\right)}{T_{t}}\right)\right\}$$

where $P_{Q_{\left(x,x^{\prime }\right)}}$ and $P_{Q_{\left(x,x^{\prime }\right)}^{inv}}$ represent the transition probabilities of the transition kernel and its inverse kernel, respectively, $Q_{\left(x^{\prime },x\right)}/Q_{\left(x,x^{\prime }\right)}$ represents the probability ratio when the state transitions from *x* to *x’*, and the opposite directions, $U\left(x^{\prime }\right)$ and $U\left(x\right)$, represent the Gibson energy values for the new state and the current state, respectively, which represent the temperature, which is used to minimize the energy. At the same time, we sample a random number $\delta \in \left[0,1\right]$ and make a judgment. $if\;\delta <\alpha \left(x,x^{\prime }\right)$, then accept the new state; otherwise, keep the original configuration state, and repeat this process until the convergence criterion is reached to obtain the optimal configuration of the crater distribution.

As mentioned above, this article sets the state transition as three states: birth, death, and update. We will use three transition kernels and define the probabilities corresponding to the transition kernel in Equation (16) as *P_B_*, *P_D,_* and *P_Up_*, respectively. The probability ratio $Q_{\left(x^{\prime },x\right)}/Q_{\left(x,x^{\prime }\right)}$ is represented by the ratio of the intensity parameter λ that conforms to the Poisson distribution for the number of objects *n*. The acceptance rate when choosing the birth event for the state transition of a given state can be calculated as follows:

(21) $$\alpha_{B}\;=\;\min\left\{\;1\;,\;\frac{P_{D}}{P_{B}}\cdot \frac{\lambda }{n^{*}+1}\cdot \exp\left(-\frac{U\left(x^{\prime }\right)-U\left(x\right)}{T_{t}}\right)\right\}$$

where $n^{*}$ represents the number of crater targets in the new configuration state after selecting the birth event. Similarly, the acceptance rate when choosing the death event can be calculated as follows:

(22) $$\alpha_{D}\;=\;\min\left\{\;1\;,\;\frac{P_{B}}{P_{D}}\cdot \frac{n}{\lambda }\cdot \exp\left(-\frac{U\left(x^{\prime }\right)-U\left(x\right)}{T_{t}}\right)\right\}$$

where *n* represents the number of crater targets under the current configuration.

**Figure 8:**
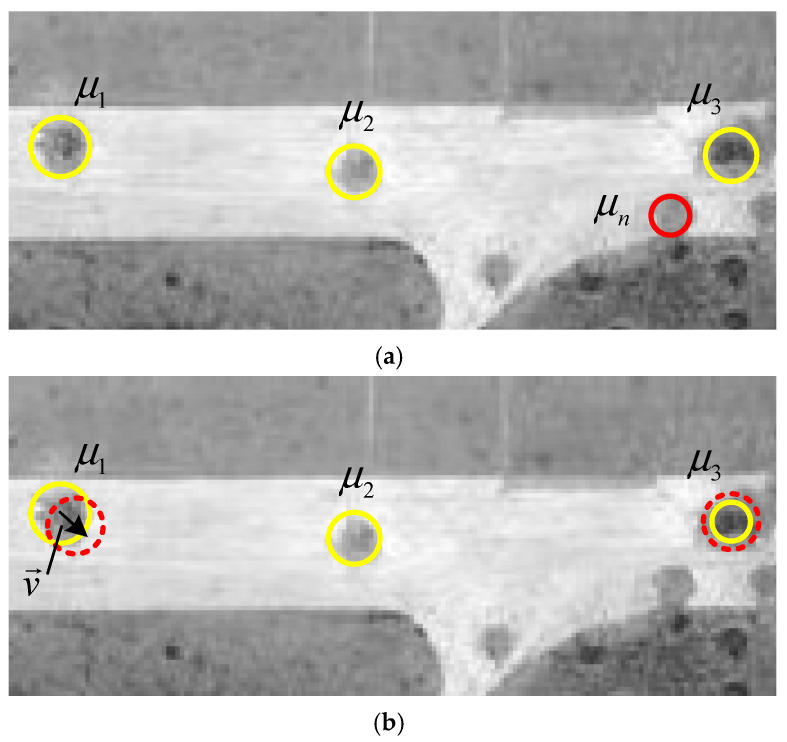
Illustration of configuration change. (**a**) The birth–death event; (**b**) the update event ($\vec{v}$ indicates horizontal movement, μ indicates the crater object).

In addition, when making state selections, sometimes a simple positional shift, size change, or other variation in a single object in the same configuration is more effective for the convergence of the entire Markov chain than birth and death events. Therefore, in addition to birth and death events, we allow for changes to the label of the selected object with a certain probability *P_Up_*. Marking changes mainly include position translation and size expansion (as shown in Figure 8b). For position changes, an object in the current configuration is randomly selected and moved a certain distance in a randomly selected direction with reference to the center of the current position. For size expansion, new values are randomly selected for randomly selected objects within a predefined range $\left[r_{\min},r_{\max}\right]$ of crater radius based on the center coordinates of the current position, and the marked energy values are updated based on the selected new values. Since all changes are set to be equally possible, the acceptance rate can be simply calculated as follows:

(23) $$\alpha_{Up}\;=\;\min\left\{\;1\;,\;\exp\left(-\frac{U\left(x^{\prime }\right)-U\left(x\right)}{T_{t}}\right)\right\}$$

To minimize the energy, the RJMCMC sampler is coupled with simulated annealing. For that reason, the parameter *T_t_* at iteration *t*, referred to as temperature, is introduced in Equation (20). The sequence of temperatures *T_t_* tends towards zero while *t* → ∞. While a logarithmic cooling schedule guarantees convergence to the global optimum, a cooling scheme based on a geometric sequence is usually used by reducing the temperature using a factor of *f_T_* in the form $T_{t}=T_{0}\cdot f_{T}^{t}$. We set the starting temperature to *T*_0_ = 100 and *f_T_* = 0.994. The optimization stops as soon as the number of objects does not change for $t=10^{4}$.

#### 2.3.3. Search Space

To reduce the computational load of the MPPs during the sampling process and avoid random searches throughout the entire image, we limit the sampling search space. We use the candidate regions obtained in Section 2.2.3 to limit the search space for crater detection. Each candidate region corresponds to a potential crater area, and the candidate regions are marked with central coordinates and radius dimensions. At the same time, in combination with the screening strategy, the interference in the background area can be reduced, the difficulty of search optimization can be lowered, and the accuracy of the detection results can be improved.

For craters extracted through the above steps, we need to determine whether they have been extracted correctly. Given the input image *I*, we assume that $\left(x_{i},y_{i}\right)$ represents the center coordinate of the extracted crater, where $x_{i}$ represents the column coordinate in the image *I*, that $y_{i}$ represents the row coordinate in the image *I*, and that $r_{i}$ represents the radius of the extracted crater. $\left({\hat{x}}_{i},{\hat{y}}_{i}\right)$ represents the labeled true crater center coordinate, where ${\hat{x}}_{i}$ and ${\hat{y}}_{i}$ represent the column coordinate and row coordinate in the image, respectively, and the corresponding true radius is expressed as ${\hat{r}}_{i}$. We impose constraints on the distance between the extracted crater center and the real crater center and the crater radius as follows [15]:

(24) $$\begin{array}{c} \sqrt{{\left(x_{i}-{\hat{x}}_{i}\right)}^{2}+{\left(y_{i}-{\hat{y}}_{i}\right)}^{2}}/\min\left(r_{i},{\hat{r}}_{i}\right)\leq \alpha_{x,y}\\ abs\left(r_{i}-\hat{r}\right)/\min\left(r_{i},{\hat{r}}_{i}\right)\leq \alpha_{r} \end{array}$$

where $\alpha_{x,y}$ and $\alpha_{r}$ represent the position and size constraint thresholds, respectively. In this experiment, we set $\alpha_{x,y}=\alpha_{r}=1$. If the extracted craters satisfy the constraints, they are considered to be correctly extracted craters.

Finally, considering the irregularity of the shape of an actual crater area, representing the crater with only a fixed shape, such as a circle or rectangle, would impose a significant deviation, which would affect the accuracy of the assessment of the functional damage effect. In the target region detected in Section 2.3, based on the light and dark difference between the grayscale values of the inner and outer areas of the crater, we use the classical active contour algorithm CV [44] to achieve a fine extraction of the crater areas. Finally, the crater extraction results are obtained by screening based on the roundness and area size of the binary extraction results.

## 3. Results

In this section, we will evaluate the performance of the crater extraction method proposed in this paper on test images (including real post-damage images of airport areas, simulated post-damage images, and post-damage images of non-airport areas). In order to further highlight the effectiveness of our proposed method, we will also conduct comparative experiments with the representative algorithm method [12] and the method utilized in [15]. We must take into account that the method in [15] is designed to extract craters within runways. Therefore, the global extraction results of the method proposed in this paper are compared with the method in [12], and the in-runway extraction results of the method proposed in this paper are compared with the method in [15]. In this paper, all the experiments were performed in MATLAB 2021b using the Windows 10 operating system, and the configuration of the computer was Intel Core I7-8750H CPU @2.20 GHz 16 GB RAM. Meanwhile, the test datasets, evaluation metrics, and experimental results used in this paper will be introduced and analyzed in detail in the following subsections.

### 3.1. Datasets

**Figure 9:**
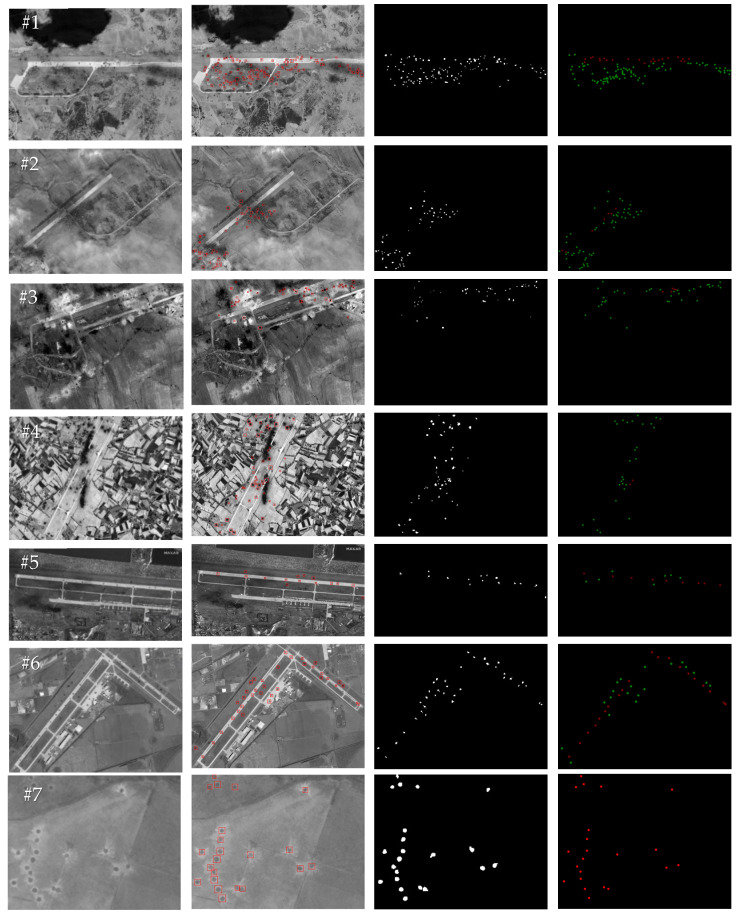

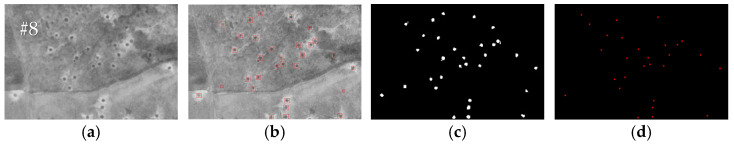
Global crater extraction results by the proposed method. (**a**) Original images; (**b**) global crater extraction results; (**c**) binary crater extraction results; (**d**) ground truth (the craters inside the runway are marked in red, the craters outside the runway are marked in green).

The crater extraction method designed in this paper mainly focuses on optical remote sensing images of the damaged airport area. The main forms of damage presented in the post-damage image are the crater areas located around the runways (inside and outside). At the same time, considering the scalability of the crater extraction method proposed in this paper and its suitability for different application scenarios, the selection of test images in this article also takes into account post-damage images of non-airport areas. As shown in Figure 9a, in this article, four remote sensing images, #1–#4, of craters distributed around airport targets after these targets were attacked, two simulated remote sensing images, #5–#6, of craters distributed around airport targets after these targets were attacked, and two remote sensing images, #7–#8, of craters distributed around non-airport targets after these targets were attacked were selected for experiments. Table 1 shows the size of each test image and the key threshold parameters used for crater detection, with the spatial resolution of the test images ranging from 2 m to 5 m. The images in Table 1 are the same as those in Figure 9a. The method in this paper only processes the corresponding grayscale image for each piece of image data tested in the experiment.

Of the selected test images, image #1 is a remote sensing image taken after the attack on Ponikve Airport in Serbia, images #2 and #3 are remote sensing images taken after the attack on Sjenica Air Base in Serbia, and image #4 is a remote sensing image taken after the attack on OBRVA Airport in Serbia; these were obtained from the internet. Images #5 and #6 were obtained from Google Earth and are remote sensing images of Chuhuiv Air Base in Ukraine and Rick Husband Amarillo International Airport in the United States, respectively. Images #7 and #8 are post-damage images obtained from the internet of non-airport targets. We simulated and generated craters placed in a random distribution around the runways in images #5 and #6. The selected test images, such as #1 and #4, have a large number of small dark interference areas, such as shadows, in the background, which makes it difficult to correctly extract the craters. At the same time, images #1 and #6 were selected for a comparative test of the model’s full-domain crater extraction against its crater extraction inside runways.

In the crater model designed in this paper, we set the parameters *f_g_*, *f_h_*, and *f_a_* to the same value of 1, the edge gradient parameter threshold to *T_g_*, the grayscale distribution consistency parameter threshold to *T_h_*, and the divergent distribution parameter threshold to *T_d_* to jointly determine the final crater extraction result. Among these, the gradient parameter threshold *T_g_* mainly affects the size of the gradient at the edge of the crater in the image. For most images, we set the parameter to 50. For images #7 and #8, we halved the threshold parameter to 25, considering factors such as the number of craters and the grayscale difference between the areas inside and outside the craters. The grayscale distribution consistency parameter *T_h_* and the divergence distribution parameter *T_d_* are set to 10 and 15, respectively. The purpose of the search range parameter *H_e_* is mainly to extract crater areas with irregular shapes and low edge roundness. For most images, we can achieve good extraction results by setting parameter *H_e_* to 0.2. For images such as #2 and #4, we raised the parameter to 0.4 because there are large areas of craters with more irregular edges. By combining the above parameter thresholds, better crater area extraction results can be achieved.

### 3.2. Evaluation Criterion

The effectiveness of the crater extraction method proposed in this paper is verified at two levels: the extraction of the global crater areas and the extraction of crater areas within a runway. In addition, the effectiveness of this method is further highlighted by comparison with other advanced methods. For the evaluation of the crater extraction results, we use the classic precision (*P*), recall (*R*), and *F-score* metrics to assess the performance of the crater extraction method proposed in this paper. The definitions of the metrics are as follows [15]:

(25) $$Precision=TP/\left(TP+FP\right)$$

(26) $$Recall=TP/\left(TP+FN\right)$$

(27) $$F_{1}=2\times Precision\times Recall/\left(Precision+Recall\right)$$

where TP represents the number of correctly extracted craters, FP represents the number of wrongly extracted craters, and FN represents the number of unsuccessfully extracted craters. All the ground truth data were obtained by manual annotation through Labelme.

### 3.3. Experimental Results and Comparison with the Existing Methods

**Figure 10:**
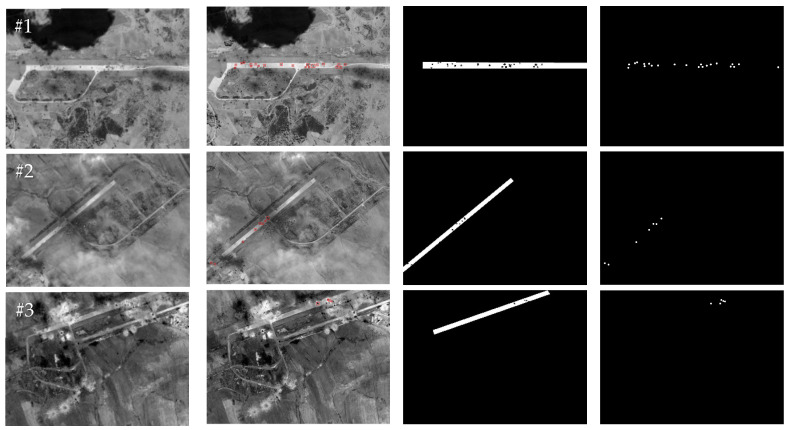

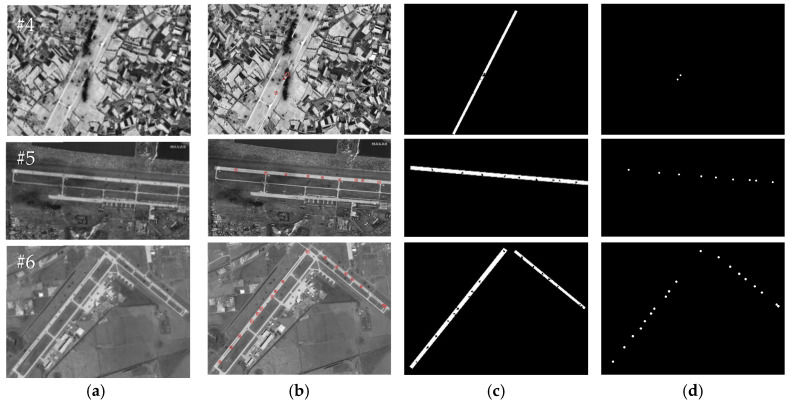
The extraction results for craters inside the runways obtained by the proposed method. (**a**) Original images; (**b**) crater extraction results; (**c**) binary crater extraction results; (**d**) ground truth.

The global crater detection and extraction results of the test images #1–#8 by the method proposed in this paper are shown in Figure 9, where the first column shows the input test images, the second column shows the crater detection results, the third column shows the binary extraction results of the crater areas, and the fourth column shows the ground truth data. The crater detection and extraction results within the runways of test images #1–#8 using the proposed method are shown in Figure 10, where the first column shows the input test images, the second column shows the extraction results for the craters located inside the runways, the third column shows the binary extraction results for the crater areas inside the runways, and the fourth column shows the ground truth data. The small, dark interference areas presented by shadows or trees in the background area of the image increase the difficulty of crater extraction (for example, the small, dark interference areas present in the backgrounds of images #1 and #4). The crater extraction method proposed in this paper first uses the overall high luminance characteristic of the runway area to extract the region of interest and reduce the interference of the background area, and then combines multiple features, such as the edge shape features of the crater area, the high gradient amplitude features of the edge, the size of the crater area, and the grayscale distribution of the area inside the crater to improve the accuracy of crater extraction. As shown in Figure 9, the method proposed in this paper achieves a relatively accurate extraction of craters distributed around airport runways with different background complexities and craters distributed around non-airport targets. As shown in Figure 10, the proposed method in this paper achieves a relatively accurate extraction of craters located within the runways.

Table 2 and Table 3, respectively, present a quantification of the extraction results of the method in this paper for all craters around the runways and the craters located inside the runways. From the quantification results in Table 2, it can be seen that the average recall *R* and precision *P* of the proposed method in this paper on all test images reached 89% and 87%, respectively, achieving an accurate extraction of all craters around the runways. Among them, the crater extraction precision *P* of image #4 was relatively low because there were a large number of dark interference areas, such as small shadows in the area adjacent to the runway, that were difficult to eliminate. However, the crater extraction recall *R* of image #4 reached 90%, indicating a relatively accurate extraction of the actual crater areas around the runway. As can be seen from the quantified results in Table 3, the average recall *R* and precision *P* of the proposed method in this paper for extracting craters located inside the runways of test images #1–#6 reached 94% and 92%, respectively, thus achieving an accurate extraction of the crater areas inside the runway. The extraction precision of image #4 was relatively low because the number of craters inside the runway in image #4 was small, and a crater adjacent to the runway was mistakenly counted inside the runway. However, the recall *R* for extracting craters located inside the runway in image #4 reached 100%, achieving an accurate extraction of the crater areas inside the runway.

**Figure 11:**
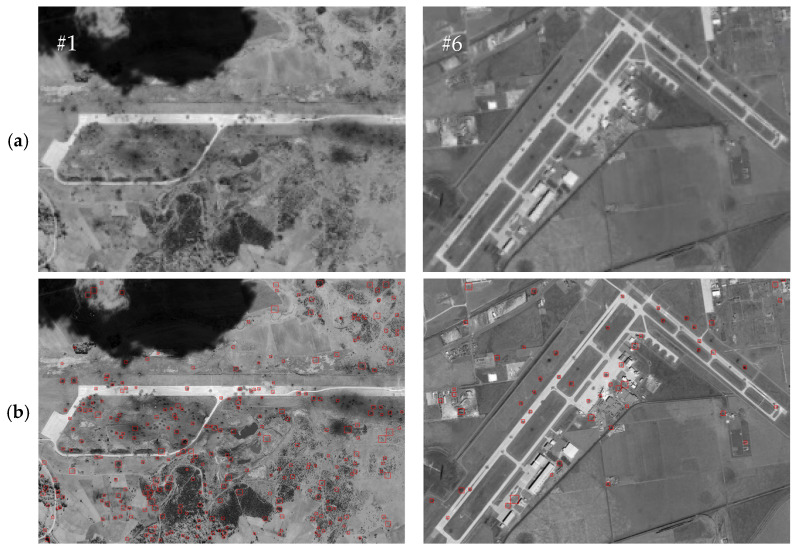

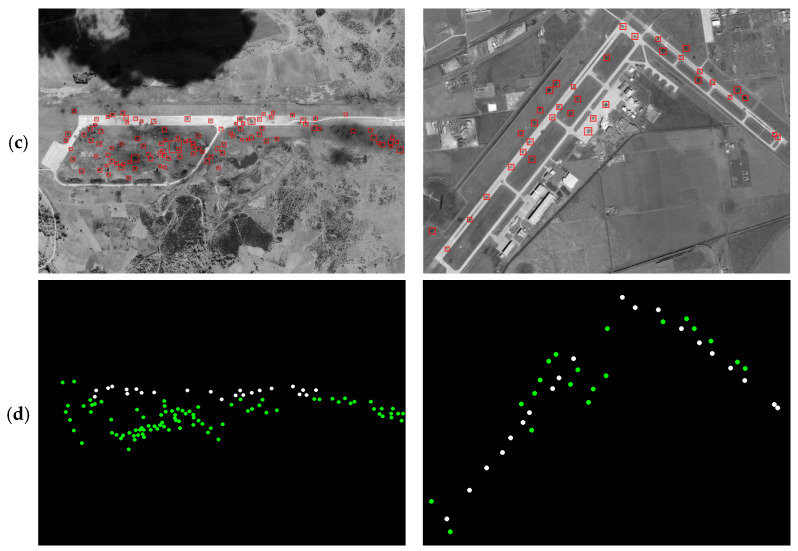
Global crater extraction results of the proposed method and the comparison method from [12] applied to images #1 and #6. (**a**) Original images; (**b**) the crater extraction results of the method from [12]; (**c**) the crater extraction results of our proposed method; (**d**) ground truth.

**Figure 12:**
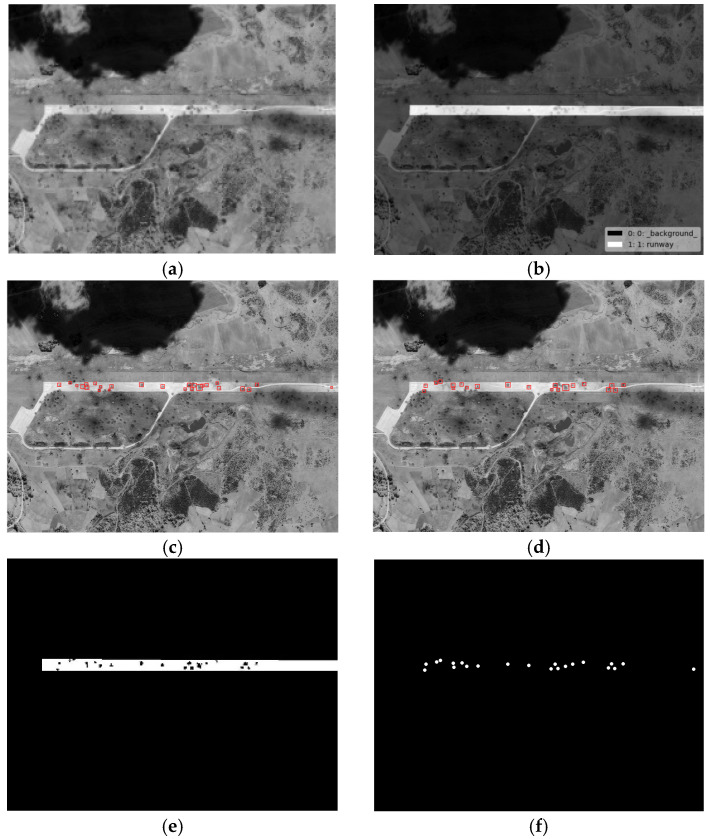
The extraction results of craters inside the runways by the proposed method and the method from [15] applied to image #1. (**a**) Original image; (**b**) the labeled runway area; (**c**) the crater extraction result of the method from [15]; (**d**) the crater extraction result of our proposed method; (**e**) the binary crater extraction result of our proposed method; (**f**) ground truth.

**Figure 13:**
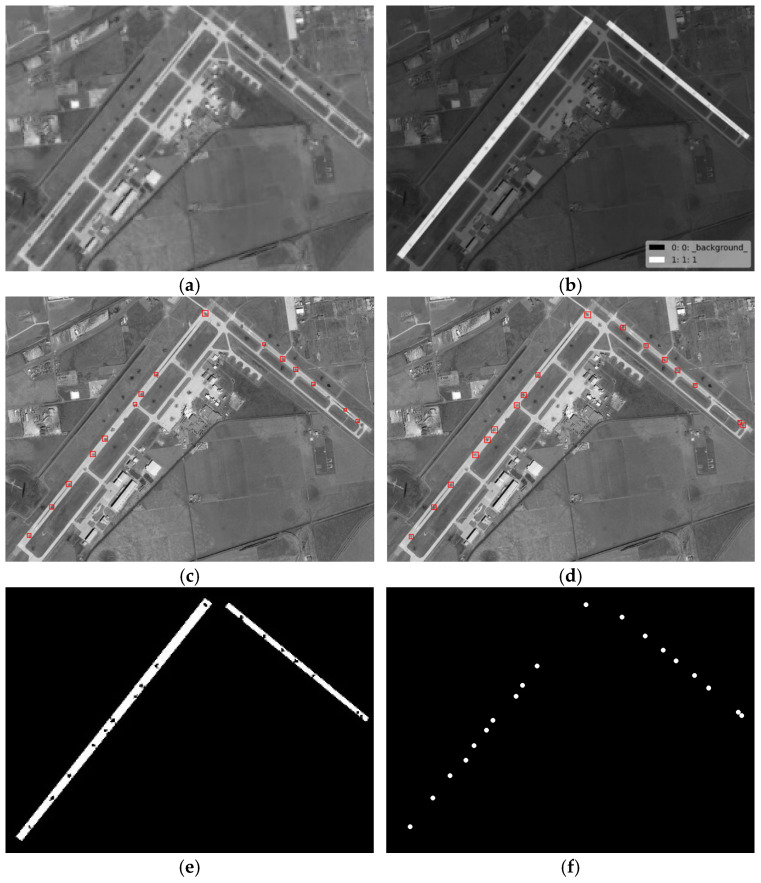
The extraction results of craters inside the runways by the proposed method and the method from [15] applied to image #6. (**a**) Original image; (**b**) the labeled runway area; (**c**) the crater extraction result of the method from [15]; (**d**) the crater extraction result of our proposed method; (**e**) the binary crater extraction result of our proposed method; (**f**) ground truth.

To further demonstrate the effectiveness of the proposed method in this paper, we conducted comparative experiments with the representative comparison algorithms defined in [12,15]. Given the small size of the crater areas, in order to present the results of the comparison experiments more clearly, we selected two images from the test images to demonstrate the effects of the comparison experiment. The craters extracted from test images #1 and #6 by the proposed method in this paper and their comparison methods are shown in Figure 11, Figure 12 and Figure 13 below. We chose image #1 because it is a real damaged image of an airport target after being attacked, and there are more interference areas in the background, so it can better demonstrate the performance of the proposed method in this paper. We chose image #6 because there are relatively fewer interference areas in the background, and the crater areas were generated through simulation. Therefore, it is more fair for the comparison experiments and can better demonstrate the performance comparison between the proposed method in this paper and the comparison methods. Among them, Figure 12 shows a comparison of the crater extraction results by our proposed method and the comparison method from [12] on images #1 and #6. Figure 12 and Figure 13, respectively, show a comparison of our method and the comparison method from [15] when extracting craters located inside the runways in images #1 and #6.

As can be seen from Figure 11, the method from [12] performs poorly in extracting craters, with a large number of small background interference areas in the extraction results, and a large number of real craters around the runway not being extracted correctly. This may be because in the method from [12], the authors set the adjacent craters as the penalty term, and the establishment of the crater model and the calculation of the edge gradient relied on a circular template. As a result, this method does not perform well at extracting craters with poor roundness. In contrast, the crater extraction method proposed in this paper achieved relatively accurate extraction for the vast majority of crater areas around the runway. As can be seen from Figure 12 and Figure 13, the method from [15] and our proposed method in this paper both achieved relatively accurate extraction of the craters located inside the runways in images #1 and #6. However, compared with the proposed method in this paper, the method from [15] has relatively more missed and false detections of real craters in the extraction results. This might be because in the method from [15], the establishment of the crater model is relatively simple, and the calculation of the model relies on a circular template. The method from [15] calculates the gradient amplitude of the edge pixels and the consistency of the grayscale distribution within the crater area with a fixed threshold for detection. Therefore, this method performs poorly at extracting crater areas with poor roundness and is prone to false detections. The crater extraction method proposed in this paper achieves a more accurate extraction of crater areas located inside a runway.

Meanwhile, Table 4 and Table 5, respectively, present the quantified extraction results of the method proposed in this paper and the comparison methods proposed by [12,15] on the test images. As shown in Table 4 and Table 5, compared with the comparison methods, the method proposed in this paper has the highest recall and extraction precision for craters located around the runway and craters located inside the runway.

## 4. Discussion

In terms of crater extraction, the presence of shadows and trees in the background of high entropy images presents small, dark interference areas, which pose a challenge to the accurate extraction of craters. This paper presents a novel coarse-to-fine two-stage method for airport crater extraction from remote sensing images based on multi-feature fusion, which achieved an accurate extraction of craters on all test images. This method fully utilizes the local features presented in high entropy images to achieve an accurate extraction of damage information. The average recall *R* and precision *P* of the method for extracting all craters around the runway reached 89% and 87%, respectively, achieving an accurate extraction of all craters around the runway. The method in this paper achieved an average recall *R* of 94% and a precision *P* of 92% for extracting craters located inside the runway, achieving an accurate extraction of craters located inside the runway. Meanwhile, the comparative experiments show that the crater extraction recall *R* and extraction precision *P* of this method are superior to those of other representative algorithms. The reason for the poor performance of the algorithm defined by [12] is that the authors set the adjacent crater regions as the penalty term, and the establishment of the crater model and the calculation of the edge gradient rely on circular templates. As a result, the algorithm does not perform well at extracting craters with poor roundness. In the method from [15], the establishment of the crater model is relatively simple, and the calculation of the crater model relies on a circular template. At the same time, the method from [15] calculates the amplitude of the edge gradient and the consistency of the grayscale distribution within the crater areas and detects it with a fixed threshold. Therefore, this method performs poorly at extracting crater areas with poor roundness and is prone to false detections. In contrast, the method proposed in this paper first uses the overall high luminance characteristics of the runway area to extract the areas of interest to reduce the interference of areas in the background, and then combines multiple features such as the edge shape characteristics of the crater area, the edge high-gradient-amplitude characteristics, the size of the crater area, and the gray distribution of the internal area of the crater to improve the accuracy of its crater extraction. Overall, the method in this paper achieves an accurate extraction of craters within airports in remote sensing images, and the recall *R* and extraction precision *P* of the craters identified are superior to those of other representative methods.

## 5. Conclusions

This paper presents a novel coarse-to-fine two-stage method for extracting airport craters from remote sensing images based on multi-feature fusion, achieving an accurate extraction of craters on all test images. The method first uses the overall high luminance features of the runway area to extract the regions of interest and reduce the interference areas in the background, and then combines multiple features such as the edge shape features of the crater area, the edge high-gradient-amplitude feature, the size of the crater area, and the gray distribution of the area inside the crater to improve the accuracy of crater extraction. Meanwhile, in steps such as edge gradient amplitude calculation, a near-region search strategy is adopted, which enhances the ability of the proposed method to accurately extract craters with irregular shapes. The experiment results show that the average recall *R* and precision *P* for extracting all craters around the runway reach 89% and 87%, respectively, and the average recall *R* and precision *P* for extracting craters located inside the runway reach 94% and 92%, respectively, achieving an accurate extraction of craters located around and inside the runways. Meanwhile, the comparative experiments show that the proposed method outperforms other representative methods in terms of both crater extraction recall and extraction precision.

In our future work, we will focus on optimizing this method to reduce the complexity and improve its operational efficiency, so that this method can offer better and faster support for the subsequent work of damage effect assessment, because, in actual combat, the rapid assessment of the target’s damage effect plays a crucial role in the development of the next strike strategy.

## Figures and Tables

**Table 1:** Image size and key parameters for crater extraction.

Test Images	Image Size (Pixels)	Gradient Threshold (*T_g_*)	Homogeneous Gray Threshold (*T_h_*)	Distribution Threshold (*T_a_*)	Search Range (*H_e_*)
#1	1051 × 801	50	10	15	0.2
#2	1145 × 830	50	10	15	0.4
#3	1140 × 831	50	10	15	0.2
#4	1130 × 806	25	10	15	0.4
#5	1024 × 545	50	10	15	0.2
#6	858 × 619	50	10	15	0.2
#7	873 × 679	25	10	15	0.2
# 8	1236 × 822	25	10	15	0.2

**Table 2:** The quantitative extraction results of all craters around the runway.

Test Images	Recall (*R*)	Precision (*P*)	F1-Score
#1	0.78	0.78	0.64
#2	0.83	0.93	0.78
#3	0.90	0.80	0.74
#4	0.90	0.68	0.63
#5	0.88	0.93	0.82
#6	0.88	0.97	0.85
#7	1.00	1.00	1.00
#8	0.96	0.87	0.84
Average	0.89	0.87	0.79

**Table 3:** The quantitative extraction results of craters inside the runways.

Test Images	Recall (*R*)	Precision (*P*)	F1-Score
#1	0.96	1.00	0.96
#2	1.00	0.88	0.88
#3	0.80	1.00	0.80
#4	1.00	0.67	0.67
#5	1.00	1.00	1.00
#6	0.90	1.00	0.90
Average	0.94	0.92	0.87

**Table 4:** The quantitative results of our proposed method and the comparison method [12].

Indices	Images	Method [12]	Our
Recall (*R*)	#1	0.44	0.78
	#6	0.58	0.88
Precision (*P*)	#1	0.17	0.78
	#6	0.42	0.97
F1-Score	#1	0.14	0.64
	#6	0.32	0.85

**Table 5:** The quantitative results of our proposed method and the comparison method [15].

Indices	Images	Method [15]	Our
Recall (*R*)	#1	0.86	0.96
	#6	0.74	0.90
Precision (*P*)	#1	0.96	1.00
	#6	0.93	1.00
F1-Score	#1	0.91	0.96
	#6	0.70	0.90

## Data Availability

The raw data supporting the conclusions of this article will be made available by the authors on request.

## Dual-Use Research Statement

Current research is limited to the field of image processing, which is beneficial for the functional assessment and recovery of the runways and does not pose a threat to public health or national security. Authors acknowledge the dual use potential of the research involving combat and confirm that all necessary precautions have been taken to prevent potential misuse. As an ethical responsibility, authors strictly adhere to relevant national and international laws about DURC. Authors advocate for responsible deployment, ethical considerations, regulatory compliance, and transparent reporting to mitigate misuse risks and foster beneficial outcomes.
